# Approach to Cataract Surgery in a Case with Microcornea and Coloboma

**DOI:** 10.22336/rjo.2025.68

**Published:** 2025

**Authors:** Bedia Kesimal, Sücattin İlker Kocamış

**Affiliations:** Ophthalmology Department, Dışkapı Yıldırım Beyazıt Training and Research Hospital, Ankara, Turkey

**Keywords:** biometry, capsule contraction syndrome, coloboma, microcornea, phacoemulsification, IOL = Intraocular lens

## Abstract

**Objectives:**

Cataract surgery in patients with microcornea and ocular coloboma poses unique surgical and postoperative challenges. The presence of a small corneal diameter, abnormal ocular anatomy, and the risk of postoperative complications require a highly individualized approach. This case highlights the difficulties encountered in biometry measurement and the development of capsular contraction syndrome following phacoemulsification surgery.

**Case report:**

A 38-year-old male with bilateral ocular coloboma, microcornea, and nystagmus presented with progressive vision loss. Phacoemulsification with intraocular lens (IOL) implantation was performed in the right eye. Preoperative planning included keratometry by corneal topography and axial length confirmation with both optical and ultrasonic biometry. Despite meticulous planning, a postoperative refractive error and rapid anterior capsular fibrosis developed. Although neodymium: YAG capsulotomy was recommended, the patient declined the intervention.

**Discussion:**

In eyes with microcornea and coloboma, accurate biometry is often hindered by structural abnormalities, increasing the risk of refractive surprises. Additionally, the risk of intraoperative complications such as capsulorhexis extension, zonular dialysis, or vitreous prolapse is heightened. Postoperatively, capsular contraction syndrome may develop rapidly, especially if subcapsular epithelial cells are not adequately removed. This case emphasizes the need for careful intraoperative management and vigilant postoperative monitoring.

**Conclusion:**

Cataract surgery in patients with microcornea and coloboma requires thorough preoperative assessment, surgical expertise, and tailored postoperative care. Awareness of potential complications such as biometric inaccuracy and capsular contraction is essential to optimize outcomes in these complex cases.

## Introduction

Congenital ocular colobomas occur due to the failure of the embryonal fissure to close during intrauterine life [1]. The incidence varies between 0.5 and 2.4 per 10,000 births [1]. The most common ocular condition accompanying eyes with coloboma is microphthalmia [1]. In these cases, nanophthalmos, optic atrophy, nystagmus, retinal detachment, myopia, glaucoma, strabismus, cataract, and orbital cyst may accompany [2].

Cataract develops at an earlier age in eyes with coloboma compared to senile cataract, and the cataract is more complicated than anticipated. The rate of complications during cataract surgery is high in these eyes [3]. This study aims to highlight the challenges that may be encountered following phacoemulsification and implantation of an intraocular lens (IOL) in patients with microcornea and coloboma.

## Case report

**Fig. 1:**
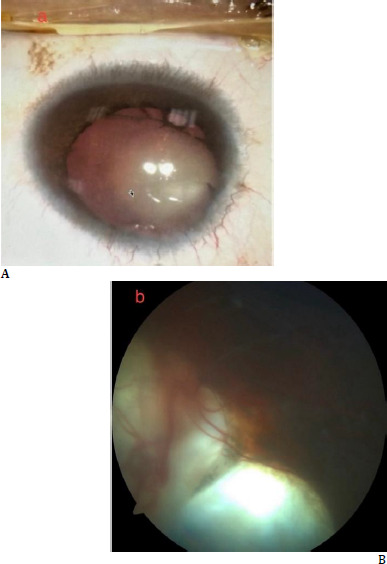
**A** Preoperative anterior segment photograph of the right eye. There was an inferior iris coloboma and nuclear cataract; **B**. Color fundus image of the right eye. There was a large retinochoroidal coloboma starting from the optic disc and spreading inferiorly; the coloboma did not involve the macula

A 38-year-old male patient presented with progressive vision loss in the right eye. He had no known systemic diseases. The best-corrected visual acuity was 1.1 logMAR in the right eye and 0.2 logMAR in the left eye. Autorefractometry revealed a refractive error of -4.75 (-0.75 axis 12) D in the right eye and -5.00 D in the left eye. Intraocular pressure was within normal limits in both eyes. Anterior segment examination revealed microcornea (horizontal corneal diameter was measured as 7.57 mm on corneal topography - Sirius, CSO, Florence, Italy), grade 3 nuclear cataract, inferior iris coloboma, nystagmus, and exotropia in the right eye (**Fig. 1A**). In contrast, the left eye showed nucleocortical cataract, inferior iris coloboma, and nystagmus [4]. Fundus examination revealed bilateral choroidal coloboma extending from the optic nerve head inferiorly, not involving the macula (**Fig. 1B**). Our patient’s retinochoroidal coloboma in both eyes was type 2 according to Ida Mann’s classification [5].

**Fig. 2:**
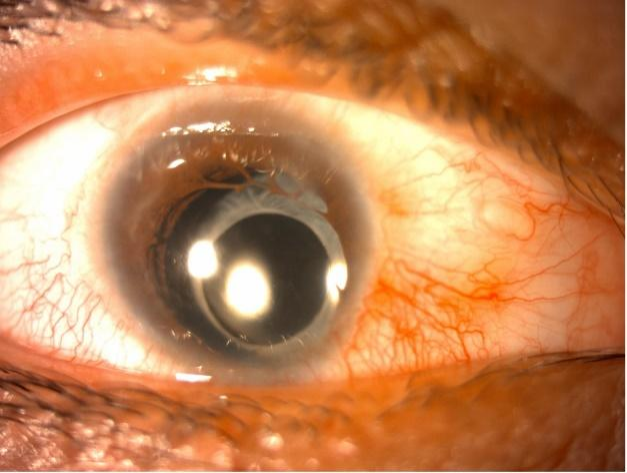
Anterior segment photograph of the right eye in postoperative month 4. A development of anterior capsular dense fibrosis and contraction was observed

Phacoemulsification surgery was planned for the right eye. Given the patient’s age and the myopia of -5.00 D in the left eye, a postoperative target of -1.00 D myopia was set. Since biometers may make incorrect measurements in eyes with microcornea, keratometry values were measured with a corneal topography. Additionally, axial length measurements were checked with an ultrasonic biometer (Aviso Quantel, Toshiba, Tokyo, Japan). The keratometry values obtained from the topography were engaged in both the Lenstar LS 900 optical biometer (Haag-Streit, Mason, Ohio, USA) and the ultrasonic biometer. Both ultrasonic and optical biometry measurements were considered, and IOL power was selected based on the SRK-T formula. Due to the microcornea, the main incision was made as a scleral tunnel, while the side ports were made using precise corneal incisions. To minimize endothelial cell damage, the soft-shell technique was employed. The nucleus was fragmented using the stop-and-chop technique. A foldable hydrophobic acrylic monobloc IOL was implanted into the capsular bag, completing the surgery. The scleral and conjunctival incisions were sutured. After administering antibiotics into the anterior chamber, the surgery was concluded. No intraoperative complications were encountered. Postoperatively, the patient was found to have -5.00 D myopia. During follow-up, fibrosis of the anterior capsule was observed. At the 4-month postoperative visit, the fibrosis had progressed, and the capsulorhexis opening had narrowed (**Fig. 2**). YAG laser capsulotomy was recommended for the anterior capsule; however, the patient declined the procedure due to potential complications.

## Discussion

In our daily practice, ocular colobomas with cataracts are among the rare ocular conditions we encounter. We aimed to present this case to emphasize the considerations we need to be aware of during and after cataract surgery in these patients.

In eyes with coloboma, the accompanying ocular findings are crucial in determining the surgical technique for cataract surgery [4]. Manual small incision cataract surgery is recommended for hard cataracts and small corneas (less than 8 mm) [4]. This preference is due to the potential for capsulorhexis tear-outs and the difficulty of rescue maneuvers in these eyes. For softer cataracts and larger corneas, phacoemulsification surgery is advised [4]. During cataract surgery in a colobomatous eye, capsulorhexis tear-out, zonular dialysis, posterior capsule rupture, vitreous prolapse, and Descemet membrane detachment may occur [3]. Zonular dialysis may develop, especially in the localization of the iris coloboma, and it is recommended that IOL haptics should be placed perpendicular to the coloboma region when performing IOL implantation [3,4]. If there is poor pupillary dilation or an eccentric pupil, an iris hook may be required [4]. In addition, due to the risk of zonular dialysis, capsular tension rings should be available during surgery. A sulcus lens should also be prepared in case there is no strong bag support. Pupilloplasty may be considered to prevent glare and diplopia complaints after cataract surgery in patients with iris coloboma and good postoperative visual potential [4]. There are documented cases in the literature of patients requiring corneal transplantation due to corneal decompensation following cataract surgery [4]. Retinal detachment may also develop, and some authors recommend prophylactic laser treatment in the preoperative period to prevent this complication [6].

In biometry measurements, parameters such as keratometry, axial length, anterior chamber depth, and lens thickness are critical [7]. Errors in these measurements can lead to inaccuracies in IOL calculations, preventing us from achieving the targeted refractive outcome. Careful consideration is needed when calculating IOL power for patients with microcornea, as the likelihood of measurement errors is higher [8]. In our patient, inaccuracies in keratometry and anterior chamber depth due to microcornea, along with erroneous axial length measurements due to coloboma, might have led to an incorrect biometry result. In eyes with microcornea and coloboma, predicting the final effective lens position is particularly challenging due to altered anatomy or zonular instability. Advanced IOL calculation formulas, such as Barrett Universal II or Olsen, which incorporate lens position prediction, may offer better outcomes in these cases [9]. Furthermore, intraoperative aberrometry could be considered to refine IOL power selection when preoperative measurements are uncertain.

Capsular contraction syndrome may develop in some patients, particularly those with high myopia, uveitis, diabetes, pseudoexfoliation syndrome, or retinitis pigmentosa [8]. It also tends to occur in patients where the anterior subcapsular epithelial cells have not been thoroughly cleaned and in those with small capsulorhexis [10]. To prevent this syndrome, subcapsular epithelial cells should be meticulously removed during surgery, and the capsulorhexis should be 5.5-6 mm in diameter [10]. The choice of IOL material is also essential. This risk is higher with silicone and hydrogel lenses, but the use of hydrophobic acrylic and polymethylmethacrylate lenses has reduced this risk [9]. In our patient, we used a hydrophobic acrylic lens. We associated the rapid and intense development of capsular fibrosis in our patient with the inadequate cleaning of the anterior subcapsular epithelial cells. When capsular contraction is detected, neodymium-YAG laser capsulotomy can be performed to create radial incisions in the anterior capsule. Another option is the surgical enlargement of the anterior capsule [1,1]. If untreated, as capsular fibrosis progresses, contraction may lead to IOL displacement, causing blurred vision. In advanced stages, it can result in IOL subluxation, ciliary detachment, and ocular hypotony [1,1]. Therefore, once capsular contraction is identified, it should be treated before further progression [10].

## Conclusion

In summary, meticulous preoperative preparation is essential when planning cataract surgery in patients with ocular coloboma. Intraoperative management requires surgical experience and expertise, while postoperative follow-up is also of utmost importance.

## References

[1] Nakamura KM, Diehl NN, Mohney BG (2011). Incidence, ocular findings, and systemic associations of ocular coloboma: a population-based study. Arch Ophthalmol.

[2] Phylactou M, Matarazzo F, Day AC, Hussain B, Maurino V (2020). Cataract surgery in eyes with congenital ocular coloboma. Graefes Arch Clin Exp Ophthalmol.

[3] Sahay P, Maharana PK, Mandal S, Sinha R, Agarwal T, Sharma N, Titiyal JS (2019). Cataract surgery outcomes in eyes with chorioretinal coloboma. J Cataract Refract Surg.

[4] Li H, Lim JH, Liu J, Wong TY (2007). Towards automatic grading of nuclear cataract. Annu Int Conf IEEE Eng Med Biol Soc.

[5] Venkatesh R, Parmar Y, Chitturi SP, Mangla R, Yadav NK, Chhablani J (2023). Ocular blood vessel arrangement in choroidal coloboma. Eye (Lond).

[6] Sen AC, Kohli GM, Mitra A, Tripathi S, Shetty SB, Gupta S (2020). Pars-plana vitrectomy with phacofragmentation for hyperdense cataracts in eyes with severe microcornea and chorio-retinal coloboma: A novel approach. Indian J Ophthalmol.

[7] Ventura BV, Ventura MC, Wang L, Koch DD, Weikert MP (2017). Comparison of biometry and intraocular lens power calculation performed by a new optical biometry device and a reference biometer. J Cataract Refract Surg.

[8] Brannan SO, Kyle G (1999). Bilateral microcornea and unilateral macrophthalmia resulting in incorrect intraocular lens selection. J Cataract Refract Surg.

[9] Stopyra W, Langenbucher A, Grzybowski A (2023). Intraocular Lens Power Calculation Formulas-A Systematic Review. Ophthalmol Ther.

[10] Liu E, Cole S, Werner L, Hengerer F, Mamalis N, Kohnen T (2015). Pathologic evidence of pseudoexfoliation in cases of in-the-bag intraocular lens subluxation or dislocation. J Cataract Refract Surg.

[11] Page TP, Whitman J (2016). A stepwise approach for the management of capsular contraction syndrome in hinge-based accommodative intraocular lenses. Clin Ophthalmol.

